# A Key Role for Subiculum-Fornix Connectivity in Recollection in Older Age

**DOI:** 10.3389/fnsys.2018.00070

**Published:** 2019-01-10

**Authors:** Naomi Hartopp, Paul Wright, Nicola J. Ray, Tavia E. Evans, Claudia Metzler-Baddeley, John P. Aggleton, Michael J. O’Sullivan

**Affiliations:** ^1^Division of Neuroscience, Institute of Psychiatry, Psychology & Neuroscience, King’s College London, London, United Kingdom; ^2^Department of Psychology, Manchester Metropolitan University, Manchester, United Kingdom; ^3^Department of Epidemiology, Erasmus Medical Center, Rotterdam, Netherlands; ^4^School of Psychology, Cardiff University, Cardiff, United Kingdom; ^5^Centre for Clinical Research, University of Queensland, Brisbane, QLD, Australia; ^6^Royal Brisbane and Women’s Hospital, Brisbane, QLD, Australia

**Keywords:** aging, memory, fornix, subiculum, hippocampus, microstructure, subfields, MRI

## Abstract

Individual differences in memory during aging are associated with the microstructure of the fornix, a bidirectional tract connecting the hippocampus with the diencephalon, basal forebrain and cortex. To investigate the origin of alterations in fornix microstructure, measurement of hippocampal subfield volumes was combined with diffusion MRI and cognitive evaluation in a new sample of 31 healthy human participants aged 50–89 years. The fornix, uncinate and parahippocampal cingulum were reconstructed using diffusion MRI tractography. Episodic memory was assessed with free and cued verbal recall, visual recognition and paired associate learning tests. Recall performance was associated with fornix microstructure and hippocampal subfield volumes. Subiculum and CA1 volumes remained positively associated with fornix microstructure when controlling for other volumes. Subiculum volume was also associated with fornix microstructure independent of age. Regression analyses showed that subiculum-fornix associations explained more variation in recall than that of CA1-fornix associations. In a multivariable regression model, age and subiculum volume were independent predictors of free recall whilst fornix microstructure and CA1 volume were not. These results suggest that age-related changes in a network that includes the subiculum and fornix are important in cognitive change in healthy aging. These results match anatomical predictions concerning the importance of hippocampal – diencephalic projections for memory.

## Introduction

The decline of episodic memory is a common but variable accompaniment of aging. The underlying causes of inter-individual differences remain poorly understood. In a previous diffusion MRI study, we demonstrated that microstructure of the fornix – a bidirectional tract that links the hippocampal formation with the diencephalon, basal forebrain and cortex – was the main correlate of recollection in healthy older adults, as measured by tests of free recall (Metzler-Baddeley et al., 2011). This association is lost, however, when the fornix is damaged in incipient AD (Metzler-Baddeley et al., 2012b). At the same time, fornix microstructure accounts for variation in memory performance not associated with chronological age. As an example, fornix microstructure correlated with episodic recollection in a group of young adults in a narrow age range (Rudebeck et al., 2009).

The origin and histological basis of the fornix alterations that associate with recollection remain unknown. One possibility is that microstructural alterations arise from loss of whole neurons that project via the fornix. If so, one might expect to find structural abnormalities in gray matter regions harboring the relevant cell bodies. The hippocampus has an intimate structural relationship with the fornix: the large majority of fornix fibers either arises from or innervates the hippocampus (including the subiculum and presubiculum). Few hippocampal efferents, to targets beyond the temporal lobe, use alternative routes, with inputs to the retrosplenial cortex providing an exception (Saunders et al., 2005; Aggleton, 2012).

In the previous sample of older adults, the relationship between fornix microstructure and recall was little altered by controlling for hippocampal volume (Metzler-Baddeley et al., 2011). However, measurements were limited to the whole hippocampus. Given that the fornix is composed of topographically organized projections from specific hippocampal subfields, important relationships may not be represented by treating the hippocampus as a single structure. Furthermore, the pattern of associations with hippocampal subfields could provide insights into underlying pathophysiology of age-related variations in memory. Histological studies have shown qualitatively different patterns of neuronal density change in AD and disease-free aging, with selective involvement of CA1 in AD, in contrast to a linear loss of neuronal density in the subiculum in those free of symptoms (West et al., 1994; Csernansky et al., 2005; Mueller and Weiner, 2009; Mueller et al., 2011; Li et al., 2013).

With recent advances in methodology, hippocampal subfields can now be investigated in more detail. Atlas-based registration – derived from manual labeling of hippocampal structures in an *ex vivo* sample imaged at 0.13 mm resolution (Iglesias et al., 2015) – provides an opportunity to address such questions directly *in vivo*. The intended starting point of this study was to test the robustness of the earlier finding, in an independent sample, by replicating the association of the fornix with age-related decline in recollection. The novel objective was then to investigate the basis of this finding by parallel evaluation of hippocampal subfields. The relationships between subfield volumes and both memory performance and white matter tract microstructure were assessed. Regression models were used to determine whether specific subfields were independently associated with fornix microstructure and memory, and to establish which relationships between gray matter volume and white matter microstructure were most relevant to cognitive performance.

## Materials and Methods

### Participants

A cohort of 31 healthy human participants was recruited. Participant age ranged from 50 to 89 years (mean ± SD, 72.4 ± 10.7 years). Twenty were female. The participants had spent a mean of 14.3 ± 0.5 years in full-time education. The selection criteria were similar to the previous study (Metzler-Baddeley et al., 2011) but the samples were drawn from non-overlapping geographical populations. There was no overlap between samples. Participants were identified from the Clinical Age Research Unit database at King’s College Hospital and from respondents to advertisements placed in general practices in South London. Exclusion criteria were: neurological or major psychiatric diagnosis; previous moderate to severe head injury; a prior diagnosis of a cognitive disorder or previous self-referral for cognitive symptoms; first language other than English; contraindications to MRI (e.g. pacemaker, penetrating eye injury). Recruitment and study procedures were approved by the London-Bromley Research Ethics Committee and all participants gave informed, written consent. Participants attended for a single MRI scan session and cognitive evaluation was completed over two testing sessions, taking approximately 90 min to 2 h.

### Cognitive Test Procedures

Episodic memory was assessed with three tasks. The FCSRT (Grober et al., 1988) was used as previously (Metzler-Baddeley et al., 2011). Visual recognition memory was tested using a visual recognition task displayed on a computer display screen. In the encoding phase, participants were presented with 40 faces and asked to judge whether the face was pleasant or unpleasant. In the test phase, participants were presented simultaneously with one face that they had previously seen and a novel face and asked to select the old face (forced-choice recognition). The face pictures were taken from the CAL/PAL Face Database (Minear and Park, 2004) and modified to remove variations in background and clothing (Ebner, 2008).

To test associative memory, we developed and implemented a PAL task, in which participants learned object-location associations. Objects consisted of colored and shaded line drawings (Snodgrass and Vanderwart ‘Like’ Objects, retrieved from http://wiki.cnbc.cmu.edu/Objects; Rossion and Pourtois, 2004). Examples include drawings of a colored ball, a whistle and a carrot. Spatial locations were provided by a grid of 10 identical square boxes. The grid comprised columns of two, three, three and two boxes, contiguous but not overlapping and centered horizontally and vertically. For the task, there was a set of 10 unique objects, each allocated to a unique location or box.

Stimuli were presented using the Psychology Experiment Building Language (PEBL) (Mueller and Piper, 2014) running on a Macbook Pro (Apple Inc., Cupertino, CA, United States). Each box measured 250 × 250 pixels (56 × 56 mm on the screen). Objects filled the majority of the area of a box but with an outer margin of approximately 5 mm to the box edge. Participants were shown the same object in two separate locations, one correct and one incorrect, and asked to indicate the correct location using a cursor controlled by a touch pad mouse. The pair of objects was shown continuously until one was selected. Once an object had been selected, feedback was then given after a delay of 2 s. Following a correct choice, a green smiling face was shown in the selected location, accompanied by a pleasant sound. After an incorrect choice, a red unhappy face and unpleasant tone were presented. Following an inter-trial interval of 1 s a new object was shown in a new pair of locations (boxes).

In the initial learning phase of 10 object-location associations, the choice of correct location was a guess. Following this initial learning phase, the set of 10 object-location pairs was presented three further times. The order of objects was pseudorandomized within each set so that each object appeared once before the next repetition. The initial set and three repetitions were presented consecutively with no indication of set completion. The mean ± SD durations of sets 1–4 were 105.2 ± 39.9 s, 85.3 ± 23.0 s, 72.1 ± 14.7 s, and 66.9 ± 21.9 s. The mean ± SD trial duration was 6.9 ± 3.2 s, range 4.2–44.2 s.

Immediately following the four choice-feedback sets, participants were asked to recall the object in each box. The boxes were highlighted one at a time in pseudorandom order and participants gave verbal responses. The recall test was self-paced. Recognition was scored by summing the correct responses from the three repeated sets after the learning phase, giving a maximum score of 30 for forced choice of locations based on object recognition. Recall of object identity (naming) based on location was scored out of 10.

### Magnetic Resonance Image Acquisition

Magnetic resonance imaging was carried out using a 3T General Electric MR750 scanner (GE Healthcare, Little Chalfont, Buckinghamshire, United Kingdom) at the Clinical Research Facility, King’s College Hospital. T1-weighted images were acquired using an MPRAGE sequence and comprised 192 sagittal slices with a thickness of 1.2 mm, field of view of 270 mm, in-plane resolution of 1 mm and an acquisition matrix of 256 × 256.

Diffusion weighted images were acquired using an echo planar imaging sequence with double refocused spin echo. Diffusion-encoding gradients were applied in 60 evenly distributed spatial directions at a *b*-value of 1500 s/mm^2^ with an additional six non-weighted images. Image geometry covered the whole brain using 2.0 mm axial slices with matrix size 128 × 128 and field of view 256 × 256 mm, giving isotropic voxels of 2 × 2 × 2 mm. The participant’s head was aligned such that the intercommissural line was as close to the axial plane as possible. Acquisition was peripherally gated to the cardiac cycle, giving a sequence duration of 11–20 min, a repetition time of 100,00–141,18 ms and an echo time of 66–78 ms. The flip angle was 90° and parallel imaging was used with ASSET factor 2.

### Hippocampal Subfield Volumes

**FIGURE 1:**
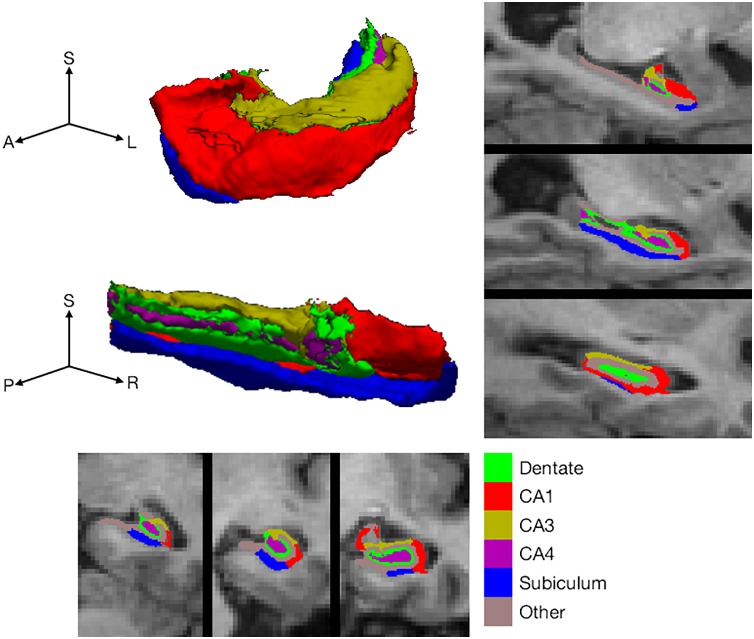
Hippocampal subfield definition. Hippocampal subfield labeling by *FreeSurfer* in a single, selected participant, illustrating the subfields included for analysis. Labels are shown on representative coronal **(bottom)** and sagittal slices **(right side)**. In addition, subfield labels are shown on the surface of the whole hippocampus **(top left)**. The whole hippocampal surface is shown from two perspectives with the three axes labeled as ***S*** for superior (or dorsal), ***A*** for anterior (or rostral), ***P*** for posterior (or caudal), ***L*** for left and ***R*** for right.

Volumetric T1-weighted images were analyzed with *FreeSurfer* (version 6.0, beta release) (Pereira et al., 2014; Iglesias et al., 2015). This version of *FreeSurfer* implements an algorithm for hippocampal segmentation which uses a generative model and Bayesian inference to attach labels derived from an *ex vivo* atlas of hippocampal subfields. The *ex vivo* atlas was derived from a training set of post-mortem images acquired at 0.13 mm resolution and labeled manually. One advantage with generative models is that they can be applied to images with different contrast characteristics; the original validation was performed on both T2 and T1-weighted datasets, including Alzheimer’s Disease Neuroimaging Initiative acquisition standards, as used in this study. Each hippocampal segmentation was checked visually for concordance with hippocampal boundaries by a single rater (NH) who reviewed coronal slices through the hippocampus with and without superimposed label boundaries. Volumes were normalized to intracranial volume also extracted from *FreeSurfer*. Cornus ammonis (CA) CA1, CA2/3 (CA2 and CA3 are difficult to distinguish so were measured in combination), CA4, DG, and subiculum were selected for analysis (Figure 1). The subiculum delineation includes both dorsal and ventral subicular regions. The prosubiculum, which is at the border between CA1 and the subiculum, was not included in the segmentation algorithm. Presubiculum and parasubiculum regions were delineated by the segmentation algorithm but are sometimes considered parahippocampal regions (Agster and Burwell, 2013) and were not included in analysis. A set of subfield volumes was generated by the *FreeSurfer* labeling algorithm in each hemisphere in each individual. Left and right hemisphere volumes were combined for analysis.

### Reconstruction of Temporal Tracts

White matter tracts were reconstructed using deterministic tractography with the ExploreDTI version 4.8.5 (Leemans and Jones, 2009). Data were preprocessed using steps developed in previous studies (Metzler-Baddeley et al., 2011, 2012b). Data were first corrected for subject motion and eddy current artifact with reorientation of the gradient encoding vectors. Free water elimination (Pasternak et al., 2009) was used to correct for cerebrospinal fluid contamination, shown previously to be important in studies involving the fornix (Metzler-Baddeley et al., 2012a). Free water elimination is based on a dual tensor model with an isotropic tensor modeling the contribution of free water (cerebrospinal fluid) and a second tensor modeling tissue.

Whole brain tractography was performed using the constrained spherical deconvolution (CSD) algorithm (Jeurissen et al., 2011) as implemented in ExploreDTI with recursive calibration of the response function. This method achieves better resolution of crossing fibers than tensor-based tracking, for example at the crossing of the fornix and the anterior commissure. Streamlines were traced from 2 mm^3^ seed points in 0.5 mm steps along the peaks of the fiber orientation density function (fODF) as estimated by CSD. After each step the direction of streamline tracking was reoriented to the fODF peaks at the new location. Tracking terminated when the fiber orientation density fell below 0.1, the change in angle exceeded 45° or the length of the streamlines exceeded 500 mm. Streamlines shorter than 50 mm were discarded. Tracts reconstructions were then extracted from whole brain tractograms based on anatomically defined ROIs as described previously (Supplementary Methods in Metzler-Baddeley et al., 2012b).

The fornix was defined using a seed ROI enclosing the body of the fornix in a coronal plane positioned just caudal to where the body begins to curve ventrally. A second ROI (“AND” ROI) was drawn around the crus of the fornix in the axial plane at the inferior edge of the corpus callosum to limit selection of streamlines to those that run through both the seed and AND ROIs. Further ROIs (“NOT” ROIs) were drawn in planes anterior, posterior, superior and inferior to the limits of the fornix to exclude streamlines that run through these areas. Additional NOT ROIs were placed to remove streamlines that were obviously inconsistent with known anatomy. The fornix was reconstructed as a single tract including both hemispheres.

The UF was defined with a seed ROI drawn in a coronal plane at the anterior edge of the corpus callosum where the uncinate is easily visible. An AND ROI was drawn around the fibers of the curve of the UF where they pass superior to inferior in an axial plane near the superior border of the pons. NOT ROIs were drawn in the midsagittal plane and in the coronal plane at the anterior border of the pons (i.e., the posterior limit of the UF). Additional NOT ROIs removed anatomically inconsistent streamlines. The left and right UF were reconstructed separately.

The PHC refers to the ventral continuation of the cingulum bundle from the posterior cingulate region into the temporal lobe. By definition in this study, the dorsal limit of the PHC is marked by the point where the cingulum begins to curve rostrally toward the temporal lobe. This definition was applied using a seed ROI around the interior part of the cingulum in an axial plane at the superior edge of the pontine white matter. The dorsal part of the cingulum was excluded with a NOT ROI in a coronal plane two slices anterior to the vertical bend of the cingulum. Additional NOT ROIs were placed in the midsagittal plane and around any anatomically inconsistent streamlines (usually those extending posteriorly into the occipital lobe).

Once tract reconstruction was complete, we calculated the mean of FA and of MD for each tract by averaging the values at each point along each streamline. In addition, mean tract *f* was derived from the Free Water Elimination procedure, as described previously. Intra-rater reliability was evaluated in a randomly chosen subset of participants (*n* = 10). Intraclass correlation coefficients for all measures of all tracts were greater than 0.89.

### Statistical Analysis

Statistical analyses were carried out using SPSS (Statistical Package for the Social Sciences, IBM corporation, versions 20 to 24). Hippocampal subfield volumes were strongly correlated between homologous regions in left and right hemispheres (Pearson’s correlation coefficients all > 0.75). Therefore, the means of left and right were used in analysis. All volumes and tract measures (FA/MD/f) were checked against the normal distribution both by visual inspection of histograms and the Shapiro–Wilk test (in all cases, *p* > 0.09). For one individual, the generated volumes of total hippocampus and all subfields were markedly greater than for all other individuals (more than 2 standard deviations from the mean). This single outlier was excluded from all analyses. Raw cognitive scores (unadjusted to age-relevant normative values) were used as variables for the statistical analyses since this study aimed to investigate correlations between inter-individual variations in cognitive performance and age.

Associations between subfields, tract microstructure and memory were evaluated with multiple linear regression. Sex and years of education were included as covariates in regression models. A set of models with age as an additional covariate were also generated to allow age-correlated and age-independent associations to be evaluated separately. To test the specificity of associations between fornix microstructure and individual subfields, an additional set of regression models that included the volume of the subfield in question and the total volume of the other subfields were tested.

Regression was also used to test whether individual subfield-tract associations could account for the variance in cognitive data, indicating relevance of these associations to memory performance. Residual values were extracted from the regression of tract microstructure on the volume of a subfield of interest (having first established that the microstructure-volume association was significant), which represent the variation of tract microstructure, which is independent of the volume of subfield of interest. Associations between these residuals and performance would suggest that variation in the tract that is not shared with the subfield in question is relevant to task performance.

This approach can be extended to a direct comparison of the cognitive relevance of associations between a tract and two subfields. For example, in a situation where tract microstructure correlates with both subfield A and subfield B, the residuals of regression between subfield and tract can be used to determine whether variation occurring in parallel with subfield A or B is most relevant to change in cognition. If there were an association between memory performance and the residuals from the regression with subfield A, it would suggest that an association between tract microstructure and memory would be found even if the relationship between tract microstructure and subfield A were constant, suggesting that subfield A is not solely driving the association between the tract and performance. If, on the other hand, residuals from the regression with subfield B were uncorrelated with performance, this would be consistent with the relationship between tract microstructure and subfield B primarily accounting for variation in memory performance. To compare between correlation coefficients, Fisher’s *r*-to-*z* transformation was used, creating a *z*-statistic and a one-tailed *p*-value (the purpose was simply to determine whether one effect was bigger than another with no hypothesis about direction of effect).

No correction for multiple statistical comparisons was applied for analyses that aimed to replicate previous findings. The novel part of the study related to hippocampal subfields and Bonferroni correction was applied for the number of hippocampal subfields: five subfields were included in the analyses so that uncorrected *p* < 0.01 (0.05/5) was considered significant.

## Results

### White Matter Tract Microstructure and Memory Performance

Fornix microstructure was associated with free recall and visual recognition memory in an age-dependent manner (Table 1). There was also evidence of association between microstructure of the PHC and memory. For tracts that could be reconstructed in both hemispheres, there was some evidence of left-right asymmetry. For verbal free recall (based on FCSRT) the association with MD was greater for the left PHC than the counterpart tract on the right (Table 1), while associations between PHC and visual recognition memory were more symmetrical.

**Table 1:** Tract microstructure and episodic memory.

	FCSRT Free Recall		FCSRT Free Recall		Visual Recognition Memory		Visual Recognition Memory	
	(sex, education)		(sex, education, and age)		(sex, education)		(sex, education, and age)	
Fornix FA	0.493^∗^		0.173		0.506^∗^		0.522^∗^	
Fornix MD	-0.477^∗^		0.107		-0.424^∗^		-0.388	
Fornix *f*	0.363^∗^		-0.065		0.172		-0.078	

	**Left**	**Right**	**Left**	**Right**	**Left**	**Right**	**Left**	**Right**

PHC FA	0.439*	0.361	0.125	0.089	0.353	0.288	0.221	0.169
PHC MD	-0.641**	-0.251	-0.372*	0.087	-0.408*	-0.392*	-0.317	-0.293
PHC *f*	0.468*	0.349	0.184	0.087	0.242	0.392*	0.075	0.305
Uncinate FA	0.028	0.096	-0.045	-0.007	-0.303	0.104	-0.352	0.029
Uncinate MD	-0.322	-0.287	0.015	0.042	-0.080	-0.132	0.144	0.057
Uncinate *f*	0.097	-0.047	-0.115	-0.070	-0.174	0.057	-0.303	0.038

*Standardized β coefficients for fractional anisotropy (FA), mean diffusivity (MD) and tissue volume fraction (f) of the three temporal tracts regressed against performance in tests of episodic memory (dependent variable). All regression models included sex and education as covariates. Regression was repeated with age as a covariate. Covariates are shown in parentheses in the column headings. FCSRT, Free and Cued Selective Reminding Test; PHC, parahippocampal cingulum. ^∗^p < 0.05; ^∗∗^p < 0.005 (uncorrected for multiple comparisons).*

### Hippocampal Subfields and Memory

**FIGURE 2:**
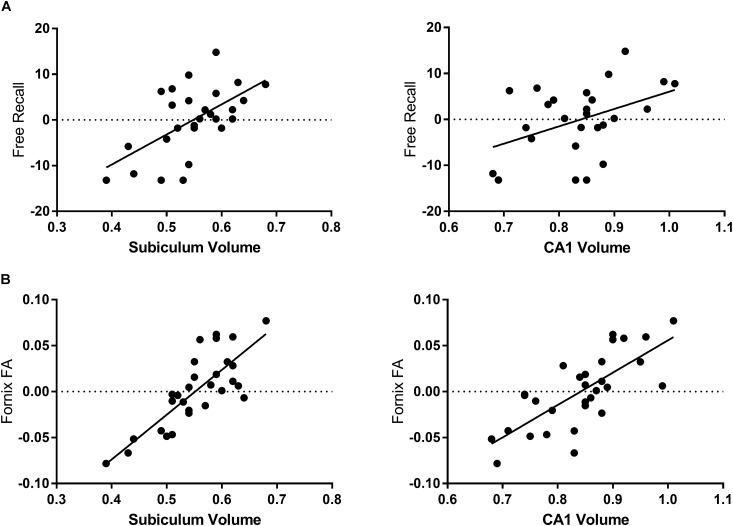
Associations of hippocampal subfields with fornix microstructure and free recall performance. Free recall is presented as the residual values of the regression of free recall and sex and education. **(A)** The volume of the subiculum is the strongest subfield correlate of age-related decline in FCSRT free recall performance. The volume of the CA1 subfield correlates less strongly. **(B)** Fornix FA correlates with the volume of all subfields measured in this study but is most strongly associated with the volume of the subiculum.

Total volume of the hippocampus was associated with free recall (β = 0.496, *p* = 0.005), visual recognition memory (β = 0.413, *p* = 0.028) and verbal recall of objects from the object-location PAL task (β = 0.45, *p* = 0.014) but not with recognition of object location. Table 2 shows the association of individual subfields with performance on memory tasks. Subiculum volume was the strongest correlate of both verbal free recall (Figure 2A) and visual recognition memory. In contrast, correct recognition of object locations in PAL was not associated with the volume of hippocampal subfields.

**Table 2:** Hippocampal subfield volumes and episodic memory.

	FCSRT Recall Free	Visual Recognition Memory	PAL Recall	PAL Recognition
Subiculum	**0.543^∗∗^**	**0.537^∗∗^**	0.316	0.054
CA1	0.389*	0.341	0.391*	0.148
Dentate gyrus	0.391*	0.384*	0.353	0.095
CA3	0.312	0.374*	0.337	0.038
CA4	0.376*	0.368	0.277	0.047

*Standardized β coefficients from multiple linear regression. All models included sex and education as covariates. FCSRT, Free and Cued Selective Reminding Test; PAL, Paired Association Learning for object-place associations. ^∗^p < 0.05; ^∗∗^p < 0.005 (uncorrected). Values that reach Bonferroni-corrected experiment wise significance are indicated in bold.*

### Hippocampal Subfield Volumes and Fornix Microstructure

The volumes of all subfields were associated with measures of fornix microstructure, with the subiculum being most strongly associated (Table 3 and Figure 2B). The pattern of association was consistent across the major microstructural measures (FA, MD, *f*), though for all subfields the highest coefficients were those with FA. (For subiculum and fornix: for FA, β = 0.82, *p* < 0.001; for MD, β = -0.64, *p* < 0.001; for *f*, β = 0.52, *p* = 0.002.) Analyses based on FA are therefore presented, though the overall pattern of results, for associations and subsequent models, were similar with MD and *f*. As subfield volumes were inter-related, regression was repeated for each individual subfield with the combined volume of the other subfields as a covariate. This analysis showed a positive association for the subiculum and CA1 (the latter not significant) after adjusting for other subfields (Table 3). Subiculum volume was associated with fornix FA independent of the volume of other subfields and, additionally, independent of age.

**Table 3:** Hippocampal subfield volumes and fornix microstructure.

	Fornix FA	Fornix FA, covarying for other subfields	Fornix FA covarying for other subfields and age
Subiculum	**0.816^∗∗∗^**	**0.701^∗∗^**	**0.597^∗∗^**
CA1	**0.736^∗∗∗^**	0.383	0.516
Dentate gyrus	**0.716^∗∗∗^**	-0.387	-0.798
CA3	**0.574^∗∗^**	-0.507	-0.576
CA4	**0.662^∗∗∗^**	-1.076	-1.190

*Standardized β coefficients for linear regression of fornix FA on subfield volume. All models are adjusted for sex and education. To adjust for volume of other subfields, the combined volume of the other four subfields was included as a covariate (middle column); age was also added (right column). FA, fractional anisotropy. ^∗^p < 0.05; ^∗∗^p < 0.005; ^∗∗∗^p < 0.0005 (uncorrected). Values that reach Bonferroni-corrected experiment wise significance are indicated in bold.*

### Subfield-Fornix Associations and Their Contribution to Memory

The analyses above show that the fornix is the principal correlate of recall performance and that hippocampal subfields – notably subiculum and CA1 – correlate with fornix microstructure. To assess the behavioral significance of these gray matter-white matter associations for memory performance, we generated measures of the variation in fornix FA that were: (i) independent of CA1 volume; and (ii) independent of subiculum volume, based on the residuals from linear regression. For verbal free recall (FCSRT Recall), the respective correlation coefficients were *r* = 0.31 (*p* = 0.012) for fornix variation independent of CA1, and *r* = 0.07 (*p* = 0.75) for variation independent of the subiculum. The difference in correlation coefficients for FCSRT Recall was significant: Fisher’s *r*-to-*z* transformation, *z* = 1.68, *p* = 0.046. The implication is that the association between subiculum and fornix FA is of significantly greater functional relevance than that between CA1 and fornix FA. For Visual Recognition Total, the respective correlation coefficients were *r* = 0.38 (*p* = 0.032) for fornix variation independent of CA1, and *r* = 0.20 (*p* = 0.29) for variation independent of the subiculum (a difference in correlation coefficients that was not significant).

### Multiple Regression Analysis of Structural Correlates of Memory

To further evaluate the relative contributions of subiculum and CA1 to performance, multiple regression was performed with FCSRT Free Recall as the dependent variable and age, fornix FA and volumes of the subiculum and CA1 as predictors. Collectively, these measures accounted for 54% of the variance in free recall. Age and subiculum volume were independent predictors of free recall (age, β = -0.64, *p* = 0.008; subiculum volume, β = 0.65, *p* = 0.025). CA1 volume and fornix microstructure were not (CA1 volume, β = -0.17, *p* = 0.52; fornix, β = -0.38, *p* = 0.22). There was no evidence of significant interaction terms, either between CA1 and subiculum volumes, or between subfield volumes and age (models not shown).

### Subfields and Other (Non-fornical) Tracts

Measurements from the three tracts (fornix, UF and PHC) displayed a degree of covariance, which was stronger for some microstructural metrics than others. For FA, there was an association between fornix and PHC but not between the fornix and UF. Consistent with this, PHC FA correlated with subfield volumes but the association was significantly weaker than that between the fornix and hippocampal subfields (fornix FA versus PHC FA for subiculum, *z* = 2.25, *p* = 0.012, based on Fisher’s *r*-to-*z* transformation). Further, FA of the fornix was the only variable independently associated with subiculum volume when FAs of all tracts were included in a multivariable regression model (model not shown).

## Discussion

This study replicates, in an independent cohort, our previous finding that the fornix is a major white matter correlate of verbal recall in older adults. By implementing a new segmentation method, based on a high-resolution *ex vivo* atlas, investigation was extended to include parallel evaluation of subfields of the hippocampus. Hippocampal subfield volumes were associated with both verbal recall and visual recognition. There was a close relationship between subiculum volume and microstructure of the fornix and this relationship was relevant to memory performance, significantly more so than the relationship between CA1 and fornix microstructure. It is possible that in older individuals, alterations in subiculum structure drive downstream degradation of fornix microstructure, which is associated with episodic memory performance. This account would fit with the cell bodies for many of the axons that project via the fornix residing in the subiculum as described in rodent and non-human primates (Christiansen et al., 2016). Further, this account is able to explain not only the decline that occurs as a corollary of aging but also inter-individual differences that are independent of chronological (if not biological) age. Subiculum volume correlated with fornix microstructure independent of age and, in a multiple regression model, both age and subiculum volume were independent predictors of verbal free recall.

The current data demonstrate the robustness of results revealing the role of the fornix in episodic memory. In an independent cohort of healthy volunteers, drawn from a different geographical population and imaged with a different MRI scanner, the current results show a similar effect size for the association of the fornix with verbal recall to previous findings (Metzler-Baddeley et al., 2011). The basis of the alterations in fornix microstructure underpinning the association with age was unclear following that original study. Although the fornix carries many hippocampal efferents, no evidence was found in the previous study of alterations in the hippocampus that were present in parallel with those in the fornix. However, that part of the analysis was limited by the techniques available at the time, to measurements of the whole hippocampus. The current results show that alterations of fornix microstructure are strongly associated with *regional* alterations within the hippocampus. When other subfields were taken into account, only the subiculum and CA1 remained positively associated with fornix FA (Table 3).

The strong and specific association with subiculum volume is highly consistent with tract-tracing studies in the macaque which show that many fornix fibers originate in the subiculum (Saunders and Aggleton, 2007) and that these subiculum fibers project to sites in the diencephalon vital for recollection (Vann et al., 2009; Carlesimo et al., 2011; Aggleton, 2012). The observed correlation is, therefore, consistent with a process that targets projections arising in the subiculum, rather than a process that directly affects white matter, as has been posited to explain alterations in white matter microstructure in other regions of the aging brain (Bartzokis, 2004). While the possibility remains that fornix fiber loss causes retrograde cell atrophy in the subiculum, studies examining the status of the hippocampus after fornix transection in non-human primates have failed to find evidence of cell loss (Daitz and Powell, 1954; Aggleton et al., 1986).

Neuropathological studies have shown that neuronal density in the subiculum declines linearly with age (West et al., 1994). In contrast, neuronal density in CA1 shows little age-related variation but marked reduction in AD. These observations contradicted an earlier view that AD was a form of ‘accelerated aging’ by demonstrating qualitative differences between AD and disease-free aging. Conversely, subiculum volume has been reported to distinguish between AD patients and those with mild cognitive impairment in a study in which CA1 volume did not (Carlesimo et al., 2015), a discrepancy which may be solved by combining imaging with pathology studies. Amyloid plaques and neurofibrillary tangles are increasingly prevalent with increasing age in the hippocampus of asymptomatic individuals (Braak and Braak, 1991), so that undetected AD pathology is one possible explanation of inter-individual variation in cognitive changes during aging. If underlying AD pathology is responsible for cognitive variation in healthy aging, it would be expected that alterations in subfield structure similar to those reported in AD, i.e., alterations in CA1, would be associated with cognitive decline in healthy aging. However, we found that associations between CA1 and fornix microstructure were less relevant to performance than those between subiculum and fornix. The regression analyses suggested that even if CA1 volume were constant, a relationship between fornix microstructure and recall would be present. Furthermore, subiculum volume was an independent predictor of verbal recall in a multiple regression model that included CA1. The pattern of results, therefore, implies mechanisms other than early neurodegeneration in CA1 in variation of recall performance by providing evidence that processes other than cell loss in CA1 are at play during aging. Instead, this pattern suggests that altered memory performance in older age is mediated by processes centered on the subiculum-fornix pathway, or alterations in hippocampal subfields that are not accompanied by volume loss. The strength of this association, between the subiculum-fornix pathway and episodic memory, may in part be explained by the convergence of input processed via the subiculum. There is evidence that the subiculum and CA1 regions are responsible for memory retrieval whilst the CA2/3 regions are involved in encoding of new memories (Suthana et al., 2015). The subiculum receives input from both the CA regions and the entorhinal cortex via CA1 as well as innervating the entorhinal cortex (Amaral and Witter, 1989; O’Mara, 2005).

The histological basis of alterations in subiculum volume in those people free of overt neurodegenerative disease is not clear. Reductions in subfield volumes do not necessarily imply neuronal cell death (Morrison and Hof, 1997). The loss of glial cells has been found to be associated with reduced gray matter volume of the hippocampus in previous studies (Willard et al., 2013) and may also contribute to alterations in white matter; for example because oligodendrocytes surround neuronal axons. In general, there is evidence that neuronal loss accompanies volume loss in the hippocampus in aging (Schuff et al., 1999). However, recent pathology studies reveal little evidence of amyloid or tau pathology, or neuronal loss, in the subiculum in disease-free aging (Wegiel et al., 2017). Mechanisms for age-related alterations independent of AD neuropathology are, however, emerging, such as accummulation of TDP43 (Yang et al., 2018), although this has not been evaluated in the subiculum specifically. Indeed, interpretation of volume changes in the subiculum is constrained by the fact that many quantitative pathology studies have failed to include or to report measurements from the subiculum (Gemmell et al., 2014). One limitation of this cross-sectional study is that the direction of effect cannot be assumed: the fornix is a bidirectional tract and carries afferents, notably from the cholinergic basal forebrain (Aggleton, 2012), which in turn could have synaptic or neurotrophic effects within the hippocampus (Knipper et al., 1994). The origin and cellular correlates of the concomitant changes that occur in subiculum and fornix in older age therefore remain unclear.

Several previous studies, using various methods, have examined hippocampal subfield-specific alterations associated in healthy aging (for review see de Flores et al., 2015). Studies of hippocampal shape change show deformations in the medial part of the hippocampal formation (Wang et al., 2003) while voxel-based morphometry studies have shown maximal changes in the superficial, inferomedial hippocampus, which also corresponds to the subiculum (Chetelat et al., 2008). One large study has shown a near linear relationship of subiculum volume with age, recapitulating the classical studies of neuronal density (West et al., 1994), while alterations in other subfields have been shown to accelerate as individuals get older (Ziegler et al., 2012). Previous imaging studies are not entirely consistent; an investigation using an earlier version of *FreeSurfer* found a pattern different to that observed here, with age effects in CA2/3 and CA4/DG (Pereira et al., 2014). The accuracy of this earlier subfield segmentation algorithm has since been questioned (de Flores et al., 2015). The use of an *ex vivo* atlas on raw data with the spatial resolution utilized in this study carries the potential for error but allowed as much anatomical information as possible to be extracted from the small hippocampal structure. An additional advantage of the new method implemented in this study is that it is based on *ex vivo* images from 15 individuals aged 60–91 years, so is well adapted to studies of aging. It is likely that delineation of subfield boundaries, particularly in the CA, is more accurate than in previous methods, but the distinction between subiculum, CA1, CA2, and CA3 remains difficult because of the lack of image contrast between them and some misclassification of voxels remains a potential limitation.

Correlations with white matter microstructure and hippocampal subfield volumes were present for verbal and visual memory and, to a lesser extent, cued recall of objects from a task based on learning object-place associations (PAL). Recognition of object location in the PAL test did not correlate with the volume of any subfield. This result is likely to be explained by a ceiling effect in performance of this task by healthy volunteers. However, there is also evidence that links neurogenesis in the DG to contextual recall (Aimone et al., 2011; Ikrar et al., 2013). Therefore, another possible factor is that performance in this task is related to cellular mechanisms, which are not represented by subiculum volume. Correct responses on the Visual Recognition task could arise from either recollection or familiarity-based mechanisms, as this task was based on a forced choice of two faces. Free recall, on the other hand, is likely to depend primarily on recollective memory. Therefore, the pattern of association overall is consistent with a general relationship with recollection. Recollection is supported by a network that includes projections carried by the fornix from the subiculum and presubiculum to diencephalic targets (Aggleton and Brown, 2006; Tsivilis et al., 2008). Critical diencephalic targets reside in the thalamus and mammillary bodies. Projections from the hippocampus to the anterior thalamic nuclei are found exclusively within the fornix. Furthermore, tracer studies in the macaque show that projections from the subicular complex to the lateral dorsal thalamic nucleus use the fornix (in contrast to entorhinal cortex projections to the lateral dorsal nucleus, which use alternative routes, Saunders and Aggleton, 2007). Therefore, there are multiple projections, to distinct thalamic nuclei that arise from the subiculum. The caudal subiculum and prosubiculum are also the major source of hippocampal projections to the mammillary body (Aggleton et al., 2005). These projections target the medial mammillary nucleus and again are routed via the fornix. In both the rat and the macaque, projections to the anterior thalamic and medial mammillary nuclei are separated along the anterior-posterior axis of the subiculum (Christiansen et al., 2016). Therefore, there are multiple parallel projection pathways between the subicular complex and distinct diencephalic targets – the medial mammillary nucleus, anterior thalamic nuclei and lateral dorsal nucleus of the thalamus, among others – all of which utilize the fornix. This convergence of multiple pathways on subiculum-fornix connectivity may explain our finding that the interdependency of structural variation in these structures is a critical factor in memory performance. This interpretation is strengthened by evidence that degeneration of the subiculum drives degeneration in connected structures such as the mammillary body and retrosplenial cortex (George et al., 2014), and that, within the thalamus, the anterior thalamic nuclei show particularly pronounced changes with aging, as does the fornix (Metzler-Baddeley et al., 2011; Fama and Sullivan, 2015).

One contrast from our previous study was the finding of associations with the PHC in healthy individuals. No consistent associations were found for the UF, which at first sight appears inconsistent with other studies of associative memory (Metzler-Baddeley et al., 2011; Alm et al., 2016). However, only total recall and recognition scores were assessed. In Alm et al. (2016) uncinate microstructure correlated with learning rate but not final accuracy (recall). Furthermore, uncinate lesions in monkeys disrupt associations between recognition and action selection (Eacott and Gaffan, 1992) but not simple object recognition.

Further investigation of alterations in subiculum structure that occur with age has the potential to highlight underlying mechanisms that might be amenable to treatment to promote cognitive health in older age. Of particular interest are those subiculum cells that project to the mammillary bodies and the anterior thalamic nuclei, as well as the neurons from both the subiculum and CA1 that reach prefrontal cortex, projections that are heavily reliant on the fornix (Aggleton, 2012; Aggleton et al., 2015) and are closely linked with episodic memory (Aggleton and Brown, 2006; Carlesimo et al., 2011). Such investigations could reveal novel treatment targets, independent of amyloid or tau accumulation in the aging brain, to ameliorate age-related cognitive decline. Identification of the critical neuronal, synaptic and glial changes that lead to subiculum atrophy, with careful correlation of quantitative imaging and neuropathology, is essential to identify new treatment targets.

## Data Availability Statement

Raw data will be made available upon request to qualified investigators, in anonymized form, in accordance with the terms of the ethical approvals of this study.

## Author Contributions

MO’S conceptualized and designed the research. PW and NR acquired the data. NH and MO’S analyzed the data and drafted the manuscript. All authors revised the manuscript for intellectual content.

## Conflict of Interest Statement

The authors declare that the research was conducted in the absence of any commercial or financial relationships that could be construed as a potential conflict of interest.

## ABBREVIATIONS

AD: Alzheimer’s disease
CA: cornu ammonis
DG: dentate gyrus
*f*: tissue volume fraction
FA: fractional anisotropy
FCSRT: Free and Cued Selective Reminding Test
MD: mean diffusivity
MRI: Magnetic Resonance Imaging
PAL: Paired Associate Learning
PHC: parahippocampal cingulum
ROI: Regions of Interest
UF: uncinate fasciculus
